# Absent Stapedial Tendon: A Case Report

**DOI:** 10.7759/cureus.71947

**Published:** 2024-10-20

**Authors:** Khalid S Alqarni, Lulwah S Alhumaidan, Saad Alenzi, Abdulrahman Alsanosi

**Affiliations:** 1 Department of Otolaryngology - Head and Neck Surgery, Prince Sultan Military Medical City, Riyadh, SAU; 2 Unaizah College of Medicine and Medical Sciences, Qassim University, Unaizah, SAU; 3 Department of Otolaryngology, Neurotology and Skull Base Surgery, King Abdullah Ear Specialist Center (KAESC), King Saud University, Riyadh, SAU; 4 Department of Otorhinolaryngology - Head and Neck Surgery, King Saud University, Riyadh, SAU

**Keywords:** absent, ear, hearing, otology, stapedius muscle

## Abstract

The stapedius muscle is a very small skeletal muscle that connects the pyramidal eminence to the stapes through the stapedial tendon. It is very rare that stapedius muscle and tendon are congenitally absent; however, this has been reported previously in the literature and the current report. Our patient was a middle-aged male who presented with hearing loss. On clinical assessment, there were no local or systemic signs and symptoms. However, after an audiological assessment that showed bilaterally mixed hearing loss, the patient was scheduled for left ear exploration and stapedectomy as a case of otosclerosis. Upon exploration, we discovered an absent stapedial muscle tendon and a non-prominent pyramid. Being aware of the stapedius muscle and tendon anomalies is substantial for surgeons performing interventions for middle ear pathologies, and more specifically while operating on patients with otosclerosis, as they showed absent stapedial tendon reflex preoperatively. Although these anomalies are not commonly seen, awareness of their existence and careful interpretation of imaging studies remain crucial.

## Introduction

Congenital anomalies of the middle ear can present either with conductive hearing loss or even accidentally detected during any surgical exploration of the middle ear [1]. The stapedius muscle is the smallest skeletal muscle. Its tendon arises from the pyramidal eminence and is inserted into the neck of stapes through the posterior surface [2]. The stapedius muscle and the tensor tympani work together to produce an acoustic reflex that has a protective role for the inner ear against harmful sounds [3]. Congenital anomalies of the stapedius muscle include ectopic, double muscles, ossificate tendon, and absent muscular unit [4]. Injury and cutting of the stapedial muscle tendon are common during otosclerosis surgeries. The incidence of absent stapedial muscle tendon has been reported to be 0.5% [1]. Owing to improved imaging modalities such as computed tomography (CT) and magnetic resonance imaging (MRI), anatomic variations or anomalies in this muscle have recently become more prominent [4]. In this study, we present a patient with a rare middle ear finding of absent stapedial tendon with regard to his clinical presentations, radiological findings, and intraoperative findings.

## Case presentation

**Figure 1:**
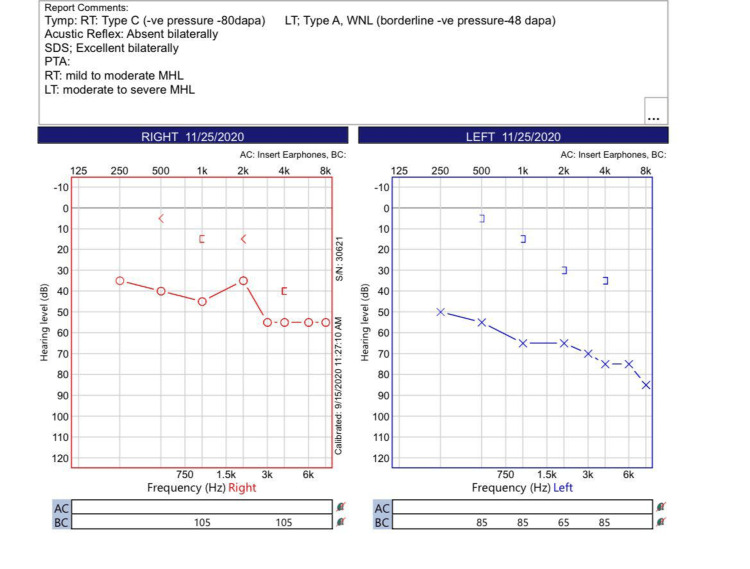
Pure-tone audiometry. PTA: pure-tone audiometry; WNL: within normal limits; SDS: speech discrimination score; MHL: mixed hearing loss; AC: air conduction; BC: bone conduction; RT: right; LT: left.

**Figure 2:**
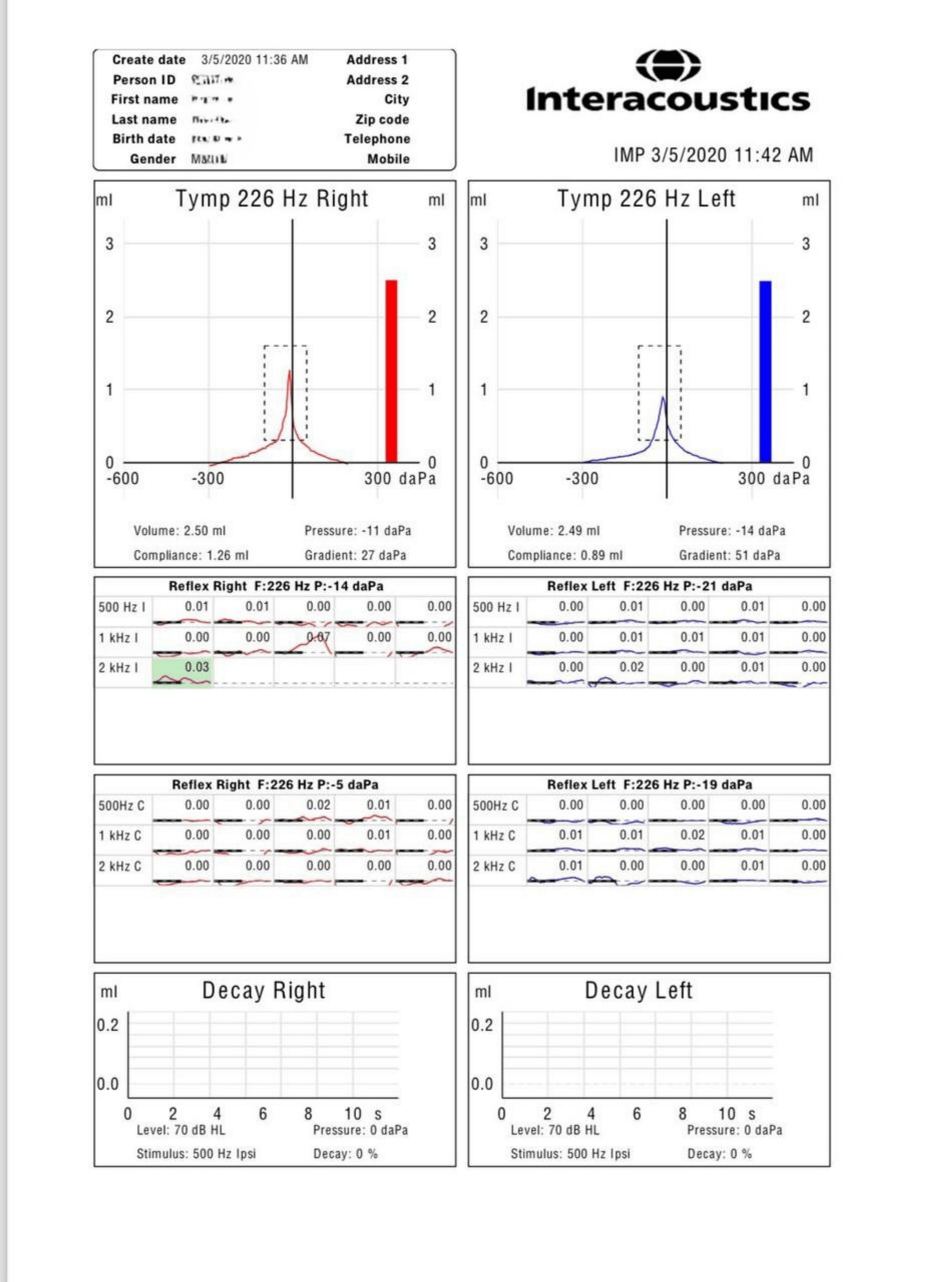
Stapedial reflex in tympanometry.

**Figure 3:**
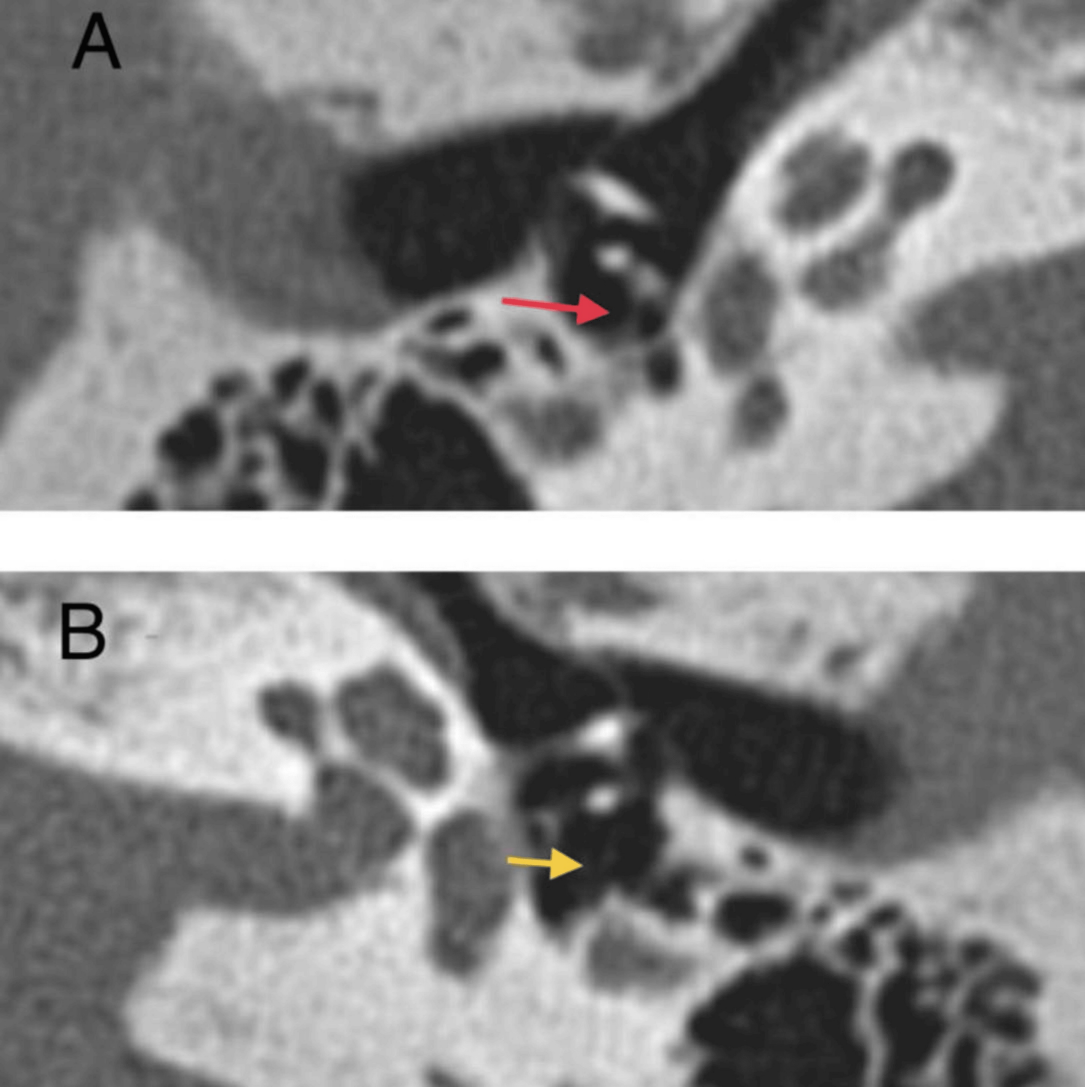
Temporal bone computed tomography (axial view). (A) The red arrow showing prominent pyramidal eminence in the right ear and the presence of a stapedial tendon. (B) The yellow arrow showing an absent stapedial tendon and non-prominent pyramidal eminence in the left ear.

A 40-year-old male, with diabetes mellitus on insulin therapy, came to the otorhinolaryngology clinic complaining of hearing loss for five years. The patient complained of bilateral progressive hearing loss. He also complained of tinnitus, mainly in the left ear. There was no otalgia, otorrhea, vertigo, or pressure sensation in the ears, and no nose or throat symptoms. The patient reported a family history of hearing loss in cousins. Further systemic clinical evaluation of the patient was unremarkable. On otoscopic examination, we observed an intact normal tympanic membrane in the right ear and an intact tympanic membrane. Weber’s test was on the left, while Rinne’s test was bilaterally negative, with a patent nasal cavity bilaterally and a normal and clear throat. Audiological studies were carried out and showed tympanometry type A bilaterally. Absent stapedial reflex bilaterally was noted (Figure 1). The pure-tone audiometry (PTA) (Figure 2) revealed a mild sloping to moderate mixed hearing loss in the right ear and a moderate sloping to severe mixed hearing loss in the left ear. Temporal computed tomography (CT) showed an absent stapedial tendon and a non-prominent pyramidal eminence in the left ear, whereas the right ear exhibited the presence of the stapedial tendon and a prominent pyramidal eminence. Bilateral otosclerotic foci were identified in the fissula ante fenestram (Figure 3). The patient was scheduled for left ear exploration and stapedectomy due to bilateral otosclerosis.

**Figure 4:**
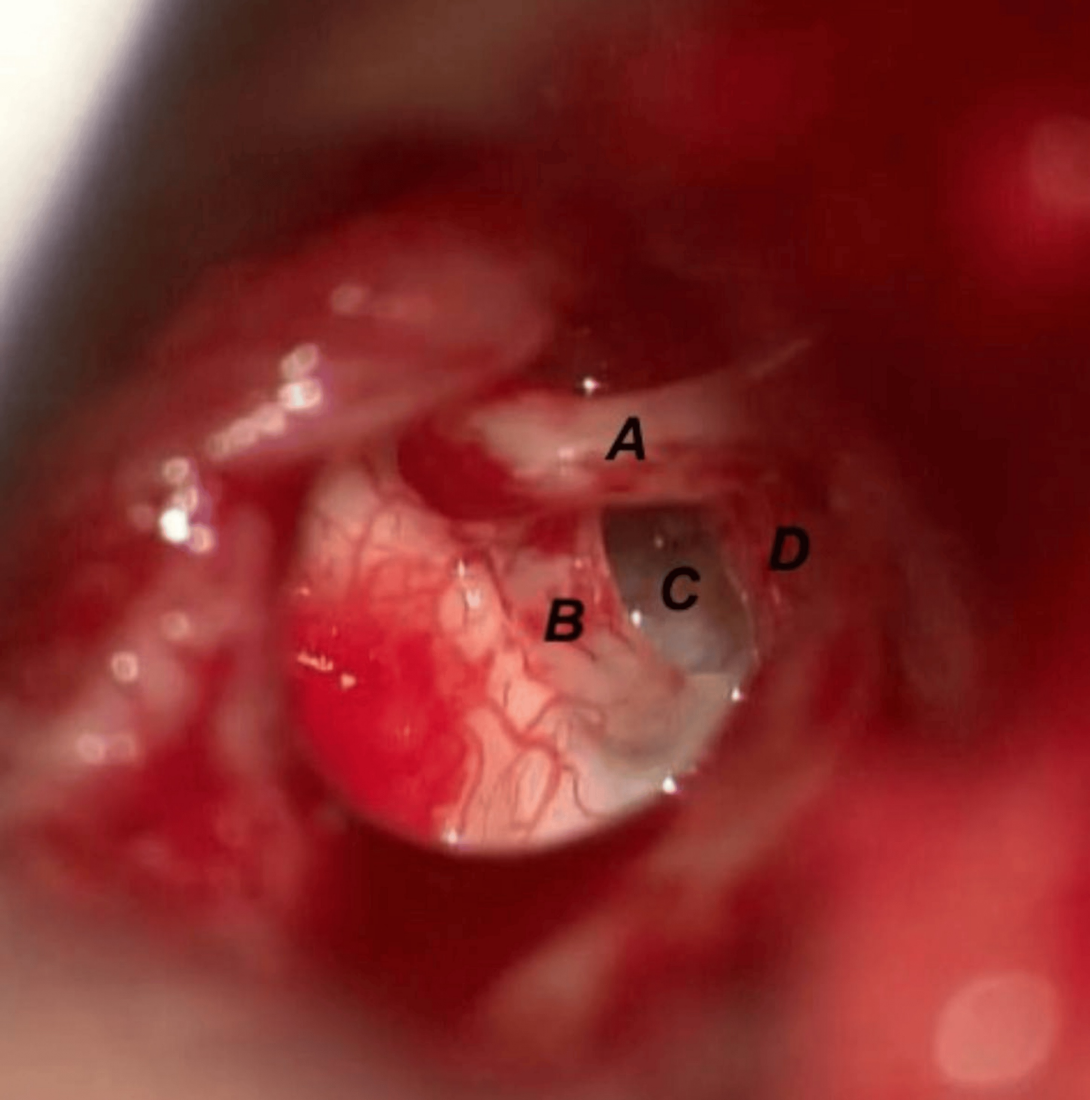
Intraoperative image. (A) Long process of incus, (B) posterior stapes crura, (C) stapes footplate, and (D) facial nerve.

In this procedure, after making an endaural incision and raising the osteomeatal flap, we observed the following: the stapes footplate was fixed; the lateral chain was mobile; the stapedial muscle tendon was absent; and the pyramid was not prominent (Figure 4).

To improve visualization, the bony overhang of the canal was removed, fully exposing the stapes footplate area. A stapedectomy was performed, creating a fenestra of 0.5 mm. A prosthesis with a 4.5 mm shaft was fitted, and fascia was harvested to reinforce the tympanic membrane. The skin was then closed to complete the procedure.

## Discussion

The stapedius muscle originates from the bundle of the posterior belly of the digastric muscle. When the digastric muscle arrives at the eminence of the mastoid process, it provides fibers of muscle through the stylomastoid opening into the tympanic cavity until it reaches the stapes neck and forms the stapedius muscle. Therefore, both muscles get innervation from the facial nerve [5]. The stapedius and tensor tympani muscles contract together for the acoustic reflex to protect ears from loud sounds and improve hearing performance during noise [5,6].

Congenital anomalies of the middle ear have previously been reported in the literature. In a previous report by Kopuz et al., there was no stapedius muscle or tendon present in the middle ear [4]. Also, the pyramidal process was not prominent, which is consistent with our report. While their patient had no other associated middle ear malformations, this has been reported in other reports. For instance, Djeric and Savic reported the absence of the stapedius tendon, as well as deformed stapes in a patient [7]. In a case series by Hough, five patients showed absent stapedius muscle [8]. Similarly, Magnuson et al. reported the absence of stapedius muscle with few tendinous fibers attached to the stapes with a prominent pyramid [9].

Moreover, Bergman et al. mentioned in their report that the stapedius muscle may be doubled or even not present, and the stapedial tendon may fail to develop. They also found ectopic stapedius muscle in 19 cases [10]. In a report by Dalmia and Behera, stapedius muscle, stapedial tendon, and pyramid were absent in two cases out of more than 500 cases who underwent stapedotomy [1]. In our report, we also found an absent stapedial tendon and a non-prominent pyramid during stapedectomy.

It has been hypothesized that the absence of stapedial muscle and tendons occurs because of non-concentrated blastema cells in the interhyale. Furthermore, another suggested reason is the failure of the muscle fascicle of the posterior belly of the digastric to reach the pyramidal eminence or the tympanic cavity [4].

## Conclusions

Absent stapedius muscle and tendon may affect the movements of the stapes and impact speech intelligibility during noise. Middle ear anomalies including absent stapedius muscle and tendon could be easily confused with other middle ear pathologies when evaluating imaging studies. Being aware of stapedius muscle and tendon anomalies is substantial for surgeons performing interventions for middle ear pathologies, especially while operating on patients with otosclerosis, as they necessitate a specific approach. Although these anomalies are not commonly seen, awareness of their existence and careful interpretation of imaging studies remain crucial. Reporting future cases in the clinical setting to the literature is recommended.
